# Phosphodiesterase 5 and Arginase Inhibitory Activities of the Extracts from Some Members of Nelumbonaceae and Nymphaeaceae Families

**DOI:** 10.3390/molecules28155821

**Published:** 2023-08-02

**Authors:** Teerapap Panklai, Nungruthai Suphrom, Prapapan Temkitthawon, Perle Totoson, Krongkarn Chootip, Xiao-Liang Yang, Hui-Ming Ge, Zhu-Jun Yao, Nattiya Chaichamnong, Kornkanok Ingkaninan, Corine Girard

**Affiliations:** 1 Center of Excellence in Cannabis Research, Faculty of Pharmaceutical Sciences and Center of Excellence for Innovation in Chemistry, Naresuan University, Phitsanulok 65000, Thailand; teerapapp62@nu.ac.th (T.P.); prapapantem@gmail.com (P.T.); 2 Université de Franche-Comté, PEPITE, 25000 Besançon, France; perle.totoson@univ-fcomte.fr (P.T.); corine.girard@univ-fcomte.fr (C.G.); 3 Department of Chemistry, Faculty of Science and Center of Excellence for Innovation in Chemistry, Naresuan University, Phitsanulok 65000, Thailand; suphrom.n1@gmail.com; 4 Department of Physiology, Faculty of Medical Science and Center of Excellence for Innovation in Chemistry, Naresuan University, Phitsanulok 65000, Thailand; krongkarnc@nu.ac.th; 5 State Key Laboratory of Coordination Chemistry, Jiangsu Key Laboratory of Advance Organic Materials, School of Chemistry and Chemical Engineering, Nanjing University, Nanjing 210023, China; yxlnmr@nju.edu.cn (X.-L.Y.); yaoz@nju.edu.cn (Z.-J.Y.); 6 State Key Laboratory of Pharmaceutical Biotechnology, Institute of Functional Biomolecules, School of Life Sciences, Nanjing University, Nanjing 210023, China; hmge@nju.edu.cn; 7 Division of Applied Thai Traditional Medicine, Faculty of Public Health, Naresuan University, Phitsanulok 65000, Thailand; orangejussamine@hotmail.com

**Keywords:** *Nymphaea pubescens*, phosphodiesterase 5, arginase, erectile dysfunction, flavonoids, lotus, water lily

## Abstract

The objectives of this study were (1) to investigate the effect of extracts from some plants in the families Nelumbonaceae and Nymphaeaceae on phosphodiesterase 5 (PDE5) and arginase, which have been used in erectile dysfunction treatment, and (2) to isolate and identify the compounds responsible for such activities. The characterization and quantitative analysis of flavonoid constituents in the active extracts were performed by HPLC. Thirty-seven ethanolic extracts from different parts of plants in the genus *Nymphaea* and *Victoria* of Nymphaeaceae and genus *Nelumbo* of Nelumbonaceae were screened for PDE5 and arginase inhibitory activities. The ethanolic extracts of the receptacles and pollens of *Nelumbo nucifera* Gaertn., petals of *Nymphaea cyanea* Roxb. ex G.Don, *Nymphaea stellata* Willd., and *Victoria amazonica* (Poepp.) Sowerby and the petals and receptacles of *Nymphaea pubescens* Willd. showed IC_50_ values on PDE5 of less than 25 μg/mL while none of the extracts showed effects on arginase. The most active extract, *N. pubescens* petal extract, was fractionated to isolate and identify the PDE5 inhibitors. The results showed that six flavonoid constituents including quercetin 3’-O-*β*-xylopyranoside (**1**), quercetin 3-methyl ether 3’-O-*β*-xylopyranoside (**2**), quercetin (**3**), 3-*O*-methylquercetin (**4**), kaempferol (**5**) and 3-*O*-methylkaempferol (**6**) inhibited PDE5 with IC_50_ values at the micromolar level.

## 1. Introduction

Erectile dysfunction (ED) is the recurrent or persistent inability to achieve or sustain a penile erection for sexual satisfaction [1]. A high prevalence of ED has been reported in eight countries: Brazil, Italy, France, China, Spain, Germany, the United States, and the United Kingdom. Italy has the highest prevalence at 48.6% of the male population, while Brazil has the lowest of these countries at 37.2% [2]. In Thailand, ED prevalence is 37.5% [3]. One of the most commonly used treatments for ED is the inhibition of the enzyme phosphodiesterase 5 (PDE5) [4]. To date, the FDA-approved oral PDE5 inhibitors that are widely available and used in the market are Sildenafil citrate (Viagra^®^), Tadalafil (Cialis^®^), Vardenafil (Levitra^®^), and Avanafil (STENDRA^®^) [5]. PDE5 is the cyclic guanosine monophosphate (cGMP)-degrading enzyme that is distributed in various tissues including the lungs, platelets, penile corpus cavernosum, smooth muscle cells, and all vascular smooth muscle cells, especially in the pulmonary vessels [6]. Inhibition of PDE5 results in raising the level of cGMP, leading to the relaxation of the vascular smooth muscle of the penile corpus cavernosum [7]. Additionally, the endothelial cells produce nitric oxide (NO), which stimulates the soluble guanylate cyclase (sGC) that changes guanosine triphosphate (GTP) to cGMP. Arginase is the enzyme implicated in ED that catalyzes the hydrolysis of L-arginine to urea and L-ornithine, while endothelium nitric oxide synthase (eNOS) converts L-arginine to L-citrulline and NO. Therefore, inhibition of arginase leads to L-arginine being more available to use for eNOS and increasing NO availability [8].

Natural products are interesting sources for drug candidates [9]. Several traditional medicinal plants in Thailand have various bioactivities, one of which improves sexual dysfunction by inhibiting the PDE5 enzyme [10,11,12,13,14,15,16]. Water lilies (*Nymphaea* spp.) are members of Thai wetland biota [17] that show interesting ethnopharmacology data. Flowers of *Nymphaea* spp. have been traditionally used for the treatment of diabetes, inflammation, jaundice, and eye disorders, and interestingly have been used as aphrodisiacs [18,19]. In this study, we explored the possible roles of some *Nymphaea* spp. and other aquatic flowering plants commonly found in Thailand in the treatment of ED by the inhibition of PDE5 and arginase enzymes. *Nymphaea* and *Victoria* genera belong to the family Nymphaeaceae. Genus *Nymphaea* contains 45–50 species which can be consumed as food and are used in traditional medicine for the treatment of various diseases. They are distributed throughout North America, Africa, Europe and Asia including Thailand [19,20]. Phytochemical studies have reported that various species of the genus *Nymphaea* contain flavonoids glycosides, alkaloids, glycosides, hydrolysable tannins, lignans, phytosterols and triterpenoid saponins [19,21]. Genus *Victoria* is distributed in the Amazonas and Chaco biogeographical regions of South America [22,23]. This genus is composed of *Victoria amazonica* (Poepp.) Sowerby, *V. cruziana* A.D. Orb. and *V. boliviana* Magdalena and L.T.Sm. Previous phytochemical studies have reported that plants in this genus contain steroids, benzenoids, chlorophylls, ketone, benzyl esters and methyl esters [22,24].

*Nelumbo* Adans. is a genus belonging to the family Nelumbonaceae. They are perennial aquatic plants of stagnant water habitats with important value in horticulture, medicine, food, religion, and culture. *Nelumbo* or lotuses contains two species, i.e., *Nelumbo nucifera* Gaertn. (The Asian lotus) and *N. lutea* Willd. (The American lotus). *N. nucifera* is distributed in many countries of North Australia, and East, South and Southeast Asia including Thailand, while *N. lutea* is distributed in North America [25,26]. Phytochemical studies have reported that the *N. nucifera* contains flavonoids, alkaloids, polysaccharides, essential oil, triterpenoids, steroids and tannins [27,28].

Some alkaloids, phenolics, and polycyclic aromatics can inhibit PDE5 activity [10,11,12,13,14,15,16,29], or arginase activity [30]. Related types of such compounds can be found in the 3 genera of aquatic plants mentioned above and therefore, the plant extracts might be good sources of the PDE5 or arginase inhibitors. The objectives of this study were to (i) investigate PDE5 and the arginase inhibitory activities of ethanolic extracts from some plants belonging to *Nelumbo*, *Nymphaea* and *Victoria* genera, (ii) isolate and identify the PDE5 and/or arginase inhibitors of the most active extract, and (iii) determine and quantitatively analyze the constituents in the various extracts.

## 2. Results

### 2.1. Phosphodiesterase-5 Inhibition of the Extracts

Thirty-seven ethanolic extracts from different parts of six species in the family of Nelumbonaceae and Nymphaeaceae were screened for PDE5 inhibitory activity. Eight extracts showed %PDE5 inhibition of more than 80% at 50 µg/mL, i.e., *N. nucifera* (receptacle and pollen), *Nymphaea* sp. (pollen), *N. cyanea* Roxb. Ex G.Don (petals), *N. stellata* Willd. (petals), *N. pubescens* Willd. (petals and receptacle), and *V. amazonica* (petals) (Table 1). These extracts were considered “active extracts” and were further tested for the concentrations that induced 50% of the maximal inhibition (IC_50_ values) (Table 2). The petals of *N. pubescens* extract had the highest IC_50_ and should be investigated for PDE5 inhibitors. This extract was fractionated to isolate and identify compounds supporting observed inhibitory activity of PDE5.

### 2.2. Arginase Inhibition of the Extracts

Thirty-seven ethanolic extracts were also screened for their arginase inhibitory activity. The results showed that all extracts had an inhibitory activity on arginase of less than 50% at 100 µg/mL, (Table 1) which is not sufficiently effective to be used as an arginase inhibitor.

### 2.3. Isolation and Identification of PDE5 Inhibitors from N. pubescens Petal Ethanolic Extract

**Figure 1:**
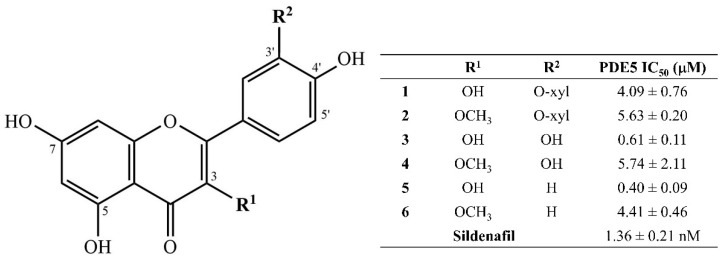
Structures of flavonoids isolated from *N. pubescens* petals and their IC_50_ values against PDE5 represented as the means ± SD from triplicate experiments. Sildenafil was used as a positive control in our assay.

From Table 2, the ethanolic extract of *N. pubescens* petals showed the highest inhibitory activity on PDE5. The extract was further fractionated by solid-phase extraction (SPE) and preparative HPLC resulting in the finding of 6 flavonoids (**1**–**6**). Four compounds were isolated and identified as quercetin 3’-O-*β*-xylopyranoside (**1**) [31], quercetin 3-methyl ether 3’-O-*β*-xylopyranoside (**2**) [31], 3-*O*-methylquercetin (**4**) [32] and 3-*O*-methylkaempferol (**6**) [33] by comparison with their spectroscopic data with the literature. In addition, the presence of quercetin (**3**) and kaempferol (**5**) in the extract was confirmed by comparison with the reference standards using HPLC analysis. The structures of these compounds and their IC_50_ values on PDE5 are shown in Figure 1. Sildenafil was used as a reference PDE5 inhibitor (IC_50_ = 1.36 ± 0.21 nM) [34].

### 2.4. The HPLC Method for Quantitative Analysis of Flavonoids in Plant Extracts

**Figure 2:**
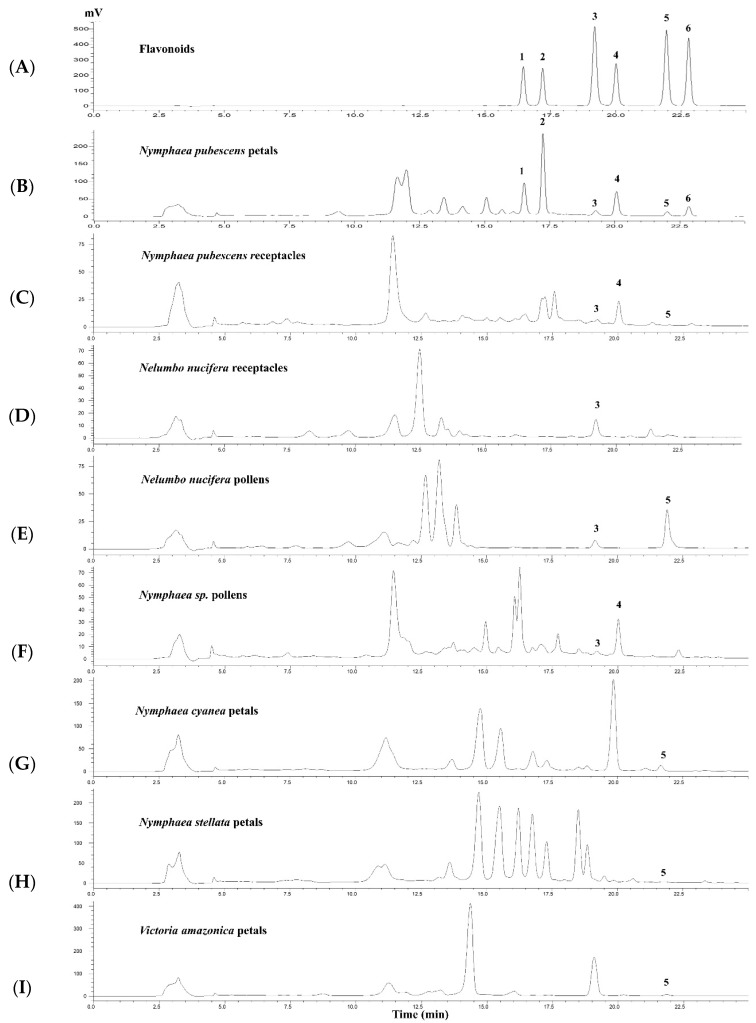
HPLC chromatograms of (**A**) mixtures of six flavonoids composed of 100 µg/mL (**1**) quercetin 3’-O-*β*-xylopyranoside, (**2**) quercetin 3-methyl ether 3’-O-*β*-xylopyranoside, (**3**) quercetin, (**4**) 3-*O*-methylquercetin, (**5**) kaempferol, and (**6**) 3-*O*-methylkaempferol, and 5 mg/mL of crude ethanolic extracts of *N. pubescens* petals (**B**), *N. pubescens* receptacles (**C**), *N. nucifera* receptacles (**D**), *N. nucifera* pollens (**E**), *Nymphaea* sp. pollens (**F**), *N. cyanea* petals (**G**), *N. stellata* petals (**H**) and *V. amazonica* petals (**I**).

The HPLC method for the quantitative determination of **1–6** in the extracts of plants in the families Nelumbonaceae and Nymphaeaceae was developed and validated. HPLC chromatograms of **1–6** and the ethanolic extract of *N. pubescens* petals are shown in Figure 2A,B. The calibration data, LOD, and LOQ values are shown in Table S1. The %RSD was less than 3% for intra-day and inter-day precision, and the range of accuracy expressed as percentage recovery is between 85.13 and 109.92% (Table S2).

### 2.5. Contents of 6 Flavonoids in Some Plant Members of Nelumbonaceae and Nymphaeaceae

HPLC analysis was applied for the detection and quantification of **1**–**6** in eight extracts (5 mg/mL) from plants belonging to the family of Nelumbonaceae and Nymphaeaceae which showed high to moderate PDE5 inhibitory activity (Table 3). From a comparison of six flavonoid constituents found in *N. pubescens* petals with other extracts, it was found that **3**, **4** and **5** were also present in other extracts whereas **1**, **2**, and **6** were only found in *N. pubescens* petal extract. Their HPLC chromatograms are shown in Figure 2B–I.

## 3. Discussion

In this study, thirty-seven ethanolic extracts from the plants of the Nelumbonaceae and Nymphaeaceae family were screened for their PDE5 and arginase inhibitory activities. This is the first time that this screening has been performed on these plants. No extract showed any significant inhibitory activity on arginase, meaning that none of the extracts was able to enhance the production of NO. This means that there was insufficient availability of NO resulting from L-arginine [8]. Conversely eight extracts showed PDE5 inhibitory activity, i.e., extracts from *N. nucifera* receptacle and pollen, *Nymphaea* sp. pollen, *N. cyanea* petals, *N. stellata* petals, *N. pubescens* petals and receptacle and *V. amazonica* petals. The petals of *N. pubescens* had the highest IC_50_ values of 6.37 ± 0.65 µg/mL whereas the pollen of *Nymphaea* sp. had the lowest IC_50_ values of 25.61 ± 2.74 µg/mL. The effect of PDE5 inhibition of these extracts have not been previously reported. The ethanolic extract of *N. stellata* leaves has shown aphrodisiac activity in male rats [35]. Neferine, which is a compound isolated from the green seed embryo of *N. nucifera*, was also reported to have a relaxant effect on rabbit corpus cavernosum tissues [36]. However, there is still no evidence that such activities related to PDE5 inhibitory effects.

Six flavonoid constituents of the *N. pubescens* petals ethanolic extract showed micromolar levels of IC_50_ against PDE5 activities. Compounds **1**, **2**, **4** and **6** were isolated and identified as components in *N. pubescens* petals for the first time. Previous studies reported that **4** was found in the *N. stellata* flowers [37], **3** and **5** were found in the *N. pubescens* petals [18,38,39], while **6** was found in the *N. stellata* flowers [40] and the *N. Alba* flowers [41] and leaves [42]. Compound **4** has been previously isolated from *Rhamnus nakaharai* Hayata (Rhamnaceae) and reported to inhibit PDE5 enzymes isolated from the lungs and hearts of male guinea pigs with an IC_50_ value of 86.9 µM [43], **3** and **5** have been previously isolated from *Anaxagorea luzonensis* and reported to inhibit PDE5 enzymes isolated from the mice lungs [14], while **1**, **2**, and **6** has not previously been reported for their effects on PDE5. Moreover, quercetin, kaempferol, and their derivatives have shown other biological activities such as antibacterial, antifungal, and anti-oxidative properties including reducing LDL oxidation, platelet aggregation, and cardiovascular complications [44].

The structure–activity relationship (SAR) on the PDE5 inhibitory activity of quercetin (**3**) and kaempferol (**5**) is depicted in Figure 1. The PDE5 inhibitory activities of **3** were 7–9 times stronger than those of **1**, **2**, and **4**, which indicated that the methyl group at position C-3 and the sugar at position C-3’ of quercetin reduced the activities. The same results were seen between **5** and **6**. The inhibitory action against PDE5 is decreased by a methyl group substitution at position C-3 of kaempferol. The PDE5 inhibitory activity of quercetin, kaempferol, and their derivatives have been reported in previous studies [14,29,45]. These compounds not only inhibit the PDE5 enzyme but also have antioxidant activity [29]. According to the review article, using PDE5 inhibitors and antioxidants in combination led to better ED without a rise in side effects [46]. However, we demonstrated the SAR of quercetin and kaempferol for the first time, which involves the substitution of methyl groups and sugars at positions C-3 and C-3’.

The HPLC method was used for the chemical characterization of the extracts in our studies, especially for the *N. pubescens* petal extract. The HPLC fingerprints of the “active” extracts were established and the quantitative determination of the six flavonoids in the extracts was validated. The HPLC chromatogram showed good separation (Figure 2). The calibration curves provided linearity range of 0.5–400 µg/mL for **1–6** with satisfactory correlation coefficient values (r^2^ = 0.9997–0.9999) (Table S1). The precisions expressed by %RSD were less than 10% and the percentage recovery was in the range of 80–120% (Table S2), which was in the acceptable range according to ICH guideline. Our results showed that this method had satisfactory sensitivity, precision and accuracy to detect and determine the flavonoids **1–6** in plant extracts. From the HPLC analysis, **2** was the major flavonoid compound in the *N. pubescens* petal extract with a concentration of 17.31 ± 0.05 mg/g. Compounds **1**, **3**, **4**, **5**, and **6** were present at much lower concentrations than **2** (2, 28, 4, 43, and 17 folds, respectively). This HPLC method can be used in a quality control process of *N. pubescens* petal extract if the extract is to be used for its health benefits relating to its PDE5 inhibitory activity. Although some HPLC systems for flavonoid analyses in other plant extracts have been reported with other plant extracts [47,48], our HPLC method was suitable for analysis of **1–6** in *N. pubescens* samples and other related spp. in our studies.

HPLC analyses of flavonoid constituents in the ethanolic extract of *N. pubescens* petals when compared with other ethanolic extracts, showed the presence of **3** in *N. nucifera* receptacle and pollen, *Nymphaea* sp. pollen and *N. pubescens* receptacle, **4** in *Nymphaea* sp. pollen and *N. pubescens* receptacle, and **5** in *N. nucifera* pollen, *N. cyanea* petals, *N. stellata petals* and *V. amazonica* petals. Paudel and Panth [28] reported that **3** and **5** were found in *N. nucifera* flower and stamen, which was similar to our results. Raja et al. [37] and Verma et al. [40] have reported that **3**, **4**, **5**, and **6** were found in *N. stellata* flower while we could observe only **5** in *N. stellata* petals. In addition, we identified the presence of flavonoids **5** in *N. cyanea* petals and *V. amazonica* petals for the first time.

## 4. Materials and Methods

### 4.1. Plant Materials

*N. pubescens* was collected from the Faculty of Pharmaceutical Science, Naresuan University, Phitsanulok, Thailand, and the other plants in the families of Nelumbonaceae and Nymphaeaceae were collected from Lotus Museum, Rajamangala University of Technology Thanyaburi, Pathum Thani, Thailand. The voucher specimens of *N. pubescens* (No. 004664), *Nymphaea* sp. (No. 05744), *N. stellata* (No. 05745), *N. cyanea* (No. 05746), *N. nucifera* (No. 05747), and *V. amazonica* (No. 05748) were kept at the Faculty of Sciences, Naresuan University, Phitsanulok, Thailand. The plants were identified by Assistant Professor Dr Pranee Nangngam, Department of Biology, Faculty of Sciences, Naresuan University.

### 4.2. Extraction and Isolation

The fresh plants were divided into leaves, petals, pollen, seed, receptacle, peduncles, and petioles, which were dried in a hot air oven at 55 °C for two days and then ground into powder. The powder was macerated with 95% ethanol for 3 days/time (two times), and then filtered and evaporated under a vacuum until dry, then stored at −20 °C until used. To isolate the PDE5 inhibitors, 9.67 g *N. pubescens* petals extract was dissolved in 100% MeOH (0.5 mL, concentration 100 mg/mL). The solution was loaded onto a solid-phase extraction (SPE) mini-column Strata C18-E (55 µm, 70 A), washed with 6 mL acetonitrile, and eluted with 0.1% formic acid in 80% water: 20% ACN for fractions 1–3 but fraction 4 was eluted with 100% acetonitrile. Fraction 4 (2.02 g) which contained flavonoids was evaporated to dryness and then purified on preparative HPLC (Gilson PLC 2020) fitted with a Kinetex EVO reverse-phase C18 column (250 × 21.2 mm, 5 µm). The solvent system used was 0.1% formic acid in 70% water (solvent A) and 0.1% formic acid in 30% acetonitrile (solvent B). The elution program (20 mL/min) was 30% B (0–25 min) and followed by a 10 min wash with 100% B and 15 min re-equilibration steps. The injection volume was 300 µL (300 mg/mL), and chromatograms were detected at 366 nm. Four pure compounds; (**1**) quercetin 3′-O-*β*-xylopyranoside (16.8 mg, Rt 16.5 min), (**2**) quercetin 3-methyl ether 3′-O-*β*-xylopyranoside (107.7 mg, Rt 17.2 min), (**4**) 3-*O*-methylquercetin (13.1 mg, Rt 20.2 min), and (**6**) 3-*O*-methylkaempferol (5.2 mg, Rt 22.9 min) were obtained. The purity of these compounds was checked by HPLC, NMR spectra, and another spectroscopic method.

### 4.3. Chemicals

Acetonitrile and methanol were of HPLC grade (VWR Chemicals, Fontenay-sous-Bois, France). Formic acid was of analytical grade (VWR Chemicals, Fontenay-sous-Bois, France). cGMP, crude snake venom (Crotalus atrox), bovine serum albumin (BSA), imidazole, ethylene glycol tetraacetic acid (EGTA), ethylenediamine tetraacetic acid (EDTA), magnesium chloride (MgCl_2_), phenyl methyl sulfonyl fluoride (PMSF), diethylaminoethyl sephadex (DEAE-Sephadex), DTT (Dithiothreitol), and tris (hydroxymethyl) aminomethane (Tris) were purchased from Sigma-Aldrich. [^3^H]-cGMP is obtained from Perkin Elmer (Boston, MA, USA). Purified liver bovine arginase 1 (1 U) of bovine arginase corresponding to the amount that was able to convert 1 µmol of L-arginine to urea and L-ornithine per minute at pH 9.5 and 37 °C) was purchase from MP Biomedicals (Illkirch-Graffenstaden, France). The (**3**) quercetin (purity > 98%, Sigma-Aldrich, St. Louis, MO, USA), and (**5**) kaempferol (purity > 90%, Sigma-Aldrich, St. Louis, MO, USA) were obtained from Sigma Chemical Company (St. Louis, MO, USA).

### 4.4. Phosphodiesterase 5 Inhibition Assay

#### 4.4.1. Sample Preparation

The extracts were tested at the final concentration of 50 μg/mL and the compounds were tested at 10 μM. All samples were dissolved in 100% DMSO and diluted with distilled water. The final concentration of DMSO was 1%. For extracts or compounds that gave >80% PDE 5 inhibition, the IC_50_ was determined.

#### 4.4.2. Enzyme Preparation

PDE5 enzymes were obtained from the transient PDE5A1 DNA transfection in human embryonic kidney 293 (HEK293) cells. These cells were homogenized using a sonicator probe and sonicate in Buffer A consisting of Tris-HCl (150 mM, pH 7.5), EDTA (6 mM), DTT (3 mM), and phenyl methyl sulfonyl fluoride (100 mM). The homogenate was centrifuged at 14,000 rpm for 20 min at 4 °C and the supernatant was used as a source of PDE5 enzymes [34].

#### 4.4.3. Experimental Protocols

A PDE5 assay was performed following the method based on a two-step radioactive procedure [49]. In the first step of the enzymatic reaction, 25 μL of extracts or solvent (5% DMSO) was added as a control to 25 μL of buffer C consisting of Tris–HCl (100 mM; pH 7.5), imidazole (100 mM), MgCl_2_ (15 mM), and BSA (1.0 mg/mL), 25 μL of EGTA (10 mM), and 25 μL of PDE5 enzymes were added together with 25 μL of [^3^H] cGMP (1 μM). This solution was then incubated at 30 °C for 10 min and then the reaction was stopped by placing it in boiling water for 1 min and cooled in ice-cold water. In the second step of the enzymatic reaction, 25 μL of snake venom (2.5 mg/mL) containing 5′-nucleotidase enzymes were added to the reaction mixture which was then incubated at 30 °C for 5 min. Then, 250 μL of Tris–HCl buffer (low salt buffer) (20 mM; pH 6.8) was added in the mixture. The reaction mixture was passed through a DEAE ion exchange resin column, and the uncharged [^3^H] guanosine was eluted 4 times with 500 µL of a low salt buffer to obtain a hydrolysis product. Finally, the scintillant cocktail was added and the radioactivity was measured using a liquid scintillation analyzer (Tris-Carb 2910 TR, Perkin Elmer). The PDE5 enzymes were standardized to have a hydrolysis activity of 20–30% of the total substrate counts. The % hydrolysis and %PDE5 inhibition were calculated by the following Equations (1) and (2).

(1) $${\%\;\mathrm{Hydrolysis}}_{\mathrm{sample}/\mathrm{control}}=[\frac{\left({\mathrm{CPM}}_{\mathrm{sample}/\mathrm{control}}\;-\;{\mathrm{CPM}}_{\mathrm{background}}\right)}{\left({\mathrm{CPM}}_{\mathrm{total}\;\mathrm{count}}\;-\;{\mathrm{CPM}}_{\mathrm{background}}\right)}\;]\times \;100$$

The CPM_sample_ is the radioactive count rate of the assay with an enzyme. CPM_background_ is the radioactive count rate of the assay but without enzyme. CPM_control_ is the radioactive count rate of the assay with enzyme but without any sample. CPM_total count_ is a count rate of 25 μL of substrate plus 2 mL of low salt buffer.

(2) $$\%\;\mathrm{PDE}\;\mathrm{inhibition}=\;\left[1-\left[\frac{{\%\;\mathrm{hydrolysis}}_{\mathrm{sample}}}{{\%\;\mathrm{hydrolysis}}_{\mathrm{control}}}\right]\right]\times \;100$$

The % hydrolysis_sample_ and % hydrolysis_control_ are the enzyme activities of the sample and solvent in the assay.

### 4.5. Arginase Inhibition Assay

#### 4.5.1. Sample Preparation

The extracts were tested at the final concentration of 100 μg/mL. All samples were dissolved in 100% DMSO and diluted with distilled water. The IC_50_ of the extracts giving >70% arginase inhibition was determined.

#### 4.5.2. Experimental Protocols

An arginase assay was performed by a spectrophotometric assay following the method in [50], based on the reaction of urea (product of arginase-catalyzed hydrolysis of L-arginine) and α-isonitrosopropiophenone with the generation of a pink imine monitored at 550 nm. The solutions were added to the 96-well microplate, in the following order: (i) buffer containing Tris-HCl (50 mM, pH 7.5) and 0.1% of bovine serum albumin (TBSA buffer) (10 μL), with or without (control) arginase (0.025 U/μL); (ii) Tris-HCl solution (50 mM, pH 7.5) containing 10 mM MnCl_2_ as a cofactor (30 μL); (iii) a solution containing an inhibitor or its solvent (as a control) (10 μL); (iv) a solution of L-arginine (pH 9.7, 0.05 M) (20 μL). The microplate was covered with a plastic sealing film and then incubated for 60 min in a 37 °C, then 120 µL of H_2_SO_4_/H_3_PO_4_/H_2_O (1:3:7) was able to stop the reaction and the microplate was left on ice for 5 min. A 10 μL volume of α-isonitrosopropiophenone (5% in absolute ethanol) was added, and the microplate was covered with an aluminium sealing film and heated in a 100 °C oven for 45 min. The microplate was kept in the dark until reading. After 5 min of centrifugation and cooling for another 10 min, the microplate was shaken for 2 min and the absorbance was read at 550 nm and 25 °C using a spectrophotometer (Synergy HT BioTeck).

### 4.6. Sample Preparation for HPLC Analysis

The concentration of extracts was prepared at 5 mg/mL and the control flavonoids consisting of compounds (**1**) quercetin 3′-O-*β*-xylopyranoside, (**2**) quercetin 3-methyl ether 3′-O-*β*-xylopyranoside, (**3**) quercetin (purity > 98%, Sigma-Aldrich), (**4**) 3-*O*-methylquercetin, (**5**) kaempferol (purity > 90%, Sigma-Aldrich), and (**6**) 3-*O*-methylkaempferol were prepared as stock solutions at 1 mg/mL with 100% methanol (HPLC grade). All samples were filtered through a 0.45 µm nylon filter before performing HPLC analysis.

### 4.7. Instrumentation and Chromatographic Conditions

HPLC analysis was performed using a Shimadzu Prominence UFLC system equipped with a Shimadzu SPD-20A UV/Vis detector, a DGU-20A3 degasser, LC-20AT liquid chromatograph, and CBM-20A communications bus module. The column used was a Phenomenex Luna C18 column (150 mm × 4.6 mm, 5 μm) connected to a Phenomenex C18 (4 mm × 3 mm, 5 μm) guard column that maintained the temperature at 40 °C. The solvent system used was 0.1% formic acid in water (solvent A) and 0.1% formic acid in acetonitrile (solvent B). The gradient system was performed by increasing the ratio of solvent B from 20% to 70% within 25 min. The flow rate was set at 0.5 mL/min, the injection volume was 10 µL, and the UV detector was detected at 366 nm.

### 4.8. Method Validation

The development of the HPLC method was validated for linearity, the limit of detection (LOD), the limit of quantification (LOQ), precision, and accuracy which were according to ICH guidelines. The concentration values of each flavonoid consisting of compounds **1**, **3**, **4**, **5**, and **6** were 0.5, 1, 5, 25, 50, 75, and 100 µg/mL, while **2** was 5, 25, 50, 75, 100, 200, and 400 µg/mL. Calibration curves were constructed from each flavonoid in triplicate (*n* = 3). The LOD and LOQ were determined using a signal-to-noise ratio of each flavonoid which was 3 for LOD and 10 for LOQ. Intra-day precision was measured in triplicate (*n* = 3), while inter-day precision was measured in triplicate for three consecutive days (*n* = 9). Precision was represented by the percentage of relative standard deviation (%RSD). The accuracy was expressed as percentage recovery by using the spiked concentration of each flavonoid in the *N. pubescens* petals. The concentration of compounds **1**, **3**, **4**, **5**, and **6** in a test solution were 3, 25, and 65 µg/mL, while that of **2** was 15, 75, and 300 µg/mL. These experiments were performed in triplicate (*n* = 3).

### 4.9. Statistical Analyses

Data were expressed as the means ± standard deviation (SD). The concentration of extracts or compounds that induced 50% of the maximal inhibition (IC_50_) was determined by fitting the original concentration–response curves using Graph Pad Prism software (version 5.0).

## 5. Conclusions

Among 37 samples from Nymphaeaceae and Nelumbonaceae families, our research found that *N. pubescens* petals had the strongest PDE5 inhibitory effect and have been re-ported for the first time. Six flavonoids with PDE5 inhibitory activity have been characterized as the constituents in the extract by the HPLC method. Compounds **1**, **2**, and **6** were found to inhibit PDE5 for the first time in this study.

## Figures and Tables

**Table 1:** Percentage of PDE5 and arginase inhibitions of ethanolic extracts from some plants in the family of Nelumbonaceae and Nymphaeaceae. The values are the means ± standard deviations (SD) from triplicate experiments.

No.	Samples	Part Used	% PDE5 Inhibitory Activity at 50 µg/mL	% Arginase Inhibitory Activity at 100 µg/mL
1	*Nelumbo nucifera*	petals	75.03 ± 1.77	17.70 ± 0.84
		pollens	85.07 ± 6.08	21.39 ± 3.82
		seeds	74.66 ± 4.39	22.25 ± 10.84
		receptacles	94.26 ± 0.74	45.30 ± 5.89
		peduncles	68.37 ± 0.50	19.98 ± 4.70
		petioles	56.71 ± 4.90	2.83 ± 5.08
		leaves	71.46 ± 0.54	21.84 ± 4.92
2	*Nymphaea* sp.	petals	78.28 ± 4.43	31.47 ± 7.71
		pollens	82.04 ± 2.06	36.91 ± 2.51
		receptacles	54.20 ± 3.26	22.07 ± 3.85
		peduncles	34.55 ± 4.37	15.62 ± 4.62
		petioles	43.54 ± 3.48	15.03 ± 2.77
		leaves	62.31 ± 4.35	30.19 ± 4.85
3	*Nymphaea cyanea*	petals	86.54 ± 1.63	31.28 ± 4.75
		pollen	73.16 ± 2.38	38.73 ± 2.17
		receptacle	62.61 ± 1.26	27.76 ± 3.66
		peduncles	34.40 ± 3.06	10.44 ± 9.32
		petioles	38.26 ± 9.82	12.09 ± 3.85
		leaves	55.53 ± 5.43	23.81 ± 3.87
4	*Nymphaea stellata*	petals	84.92 ± 1.86	28.45 ± 5.24
		pollens	38.05 ± 5.83	27.23 ± 14.29
		receptacles	46.07 ± 4.03	23.50 ± 6.07
		peduncles	13.36 ± 6.59	0
		petioles	29.17 ± 7.58	9.86 ± 4.59
		leaves	44.89 ± 7.26	21.13 ± 19.60
5	*Nymphaea pubescens*	petals	98.97 ± 0.26	39.49 ± 4.53
		pollens	74.86 ± 2.45	15.20 ± 1.87
		receptacles	82.69 ± 3.36	23.53 ± 3.57
		peduncles	58.90 ± 4.60	23.36 ± 2.52
		petioles	50.23 ± 7.61	11.96 ± 2.65
		leaves	73.26 ± 3.29	37.71 ± 3.23
6	*Victoria amazonica*	petals	86.67 ± 2.65	30.49 ± 3.22
		pollens	63.35 ± 5.45	27.61 ± 1.47
		receptacles	34.78 ± 6.41	13.30 ± 5.48
		peduncles	8.23 ± 3.92	16.87 ± 4.80
		petioles	9.16 ± 0.73	14.39 ± 5.57
		leaves	43.90 ± 3.78	21.40 ± 4.61

**Table 2:** IC_50_ values of ethanolic extracts from some plants in the family of Nelumbonaceae and Nymphaeaceae on PDE5 represented as the means ± SD from triplicate experiments.

No.	Samples	Part Used	IC_50_ Values (μg/mL)
1	*Nelumbo nucifera*	Receptacles	10.50 ± 3.65
		Pollens	14.15 ± 3.97
2	*Nymphaea* sp.	Pollens	25.61 ± 2.74
3	*Nymphaea cyanea*	Petals	8.07 ± 1.33
4	*Nymphaea stellata*	Petals	13.28 ± 0.25
5	*Nymphaea pubescens*	Petals	6.37 ± 0.65
		Receptacles	18.61 ± 4.03
6	*Victoria amazonica*	Petals	21.54 ± 4.23

**Table 3:** The flavonoid contents in some plants in the family of Nelumbonaceae and Nymphaeaceae determined by the HPLC method (*n* = 3).

Sample	Contents of the Flavonoid Constituents (Mean ± SD) (mg/g Ethanolic Extract)					
	1	2	3	4	5	6
*Nelumbo nucifera*						
Receptacles	-	-	4.55 ± 0.25	-	-	-
Pollens	-	-	2.55 ± 0.18	-	7.34 ± 0.35	-
*Nymphaea* sp.						
Pollens	-	-	3.09 ± 0.13	16.67 ± 056	-	-
*Nymphaea cyanea*						
Petals	-	-	-	-	2.71 ± 0.18	-
*Nymphaea stellata*						
Petals	-	-	-	-	0.82 ± 0.01	-
*Nymphaea pubescens*						
Petals	7.10 ± 0.10	17.31 ± 0.05	0.61 ± 0.00	4.72 ± 0.08	0.40 ± 0.00	1.04 ± 0.01
Receptacles	-	-	6.38 ± 035	8.32 ± 0.67	1.40 ± 0.19	-
*Victoria amazonica*						
Petals	-	-	-	-	1.77 ± 0.16	-

## Data Availability

The data presented in this study are available on request from the corresponding author.

## Supplementary Materials

The following supporting information can be downloaded at: https://www.mdpi.com/article/10.3390/molecules28155821/s1, Table S1: Calibration data, LOD, and LOQ of the flavonoids **1**–**6** analyzed by HPLC. Table S2: Intra- and inter-day precision and accuracy of flavonoids **1**–**6** analyzed by HPLC.

## Sample Availability

Samples of compounds **1**–**6** are available from the authors.
