# Supplementary material for: Phosphodiesterase 5 and Arginase Inhibitory Activities of the Extracts from Some Members of Nelumbonaceae and Nymphaeaceae Families
Source: Molecules. 2023 Aug 2;28(15):5821. doi: 10.3390/molecules28155821 (PMC10420992; doi:10.3390/molecules28155821)
Supplement: Supplementary file 1 [file molecules-28-05821-s001.zip › molecules-2446852-supplementary.pdf]

molecules

Supplementary Files

Phosphodiesterase 5 and Arginase Inhibitory Activities of the Extracts from Some Members of Nelumbonaceae and Nymphaeaceae Families

Teerapap Panklai [1,2], Nungruthai Suphrom [3], Prapapan Temkitthawon [1], Perle Totoson [2], Krongkarn Chootip [4], Xiao-Liang Yang [5], Hui-Ming Ge [6], Zhu-Jun Yao [5], Nattiya Chaichamnong [7], Kornkanok Ingkaninan [1,*] and Corine Girard [2]

[1] Center of Excellence in Cannabis Research, Faculty of Pharmaceutical Sciences and Center of Excellence for Innovation in Chemistry, Naresuan University, Phitsanulok 65000, Thailand; teerapapp62@nu.ac.th (T.P.); prapapantem@gmail.com (P.T.)
[2] Université de Franche-Comté, PEPITE, Besançon 25000, France; perle.totoson@univ-fcomte.fr (P.T.); corine.girard@univ-fcomte.fr (C.G.)
[3] Department of Chemistry, Faculty of Science and Center of Excellence for Innovation in Chemistry, Naresuan University, Phitsanulok 65000, Thailand; suphrom.n1@gmail.com
[4] Department of Physiology, Faculty of Medical Science and Center of Excellence for Innovation in Chemistry, Naresuan University, Phitsanulok 65000, Thailand; krongkarnc@nu.ac.th
[5] State Key Laboratory of Coordination Chemistry, Jiangsu Key Laboratory of Advance Organic Materials, School of Chemistry and Chemical Engineering, Nanjing University, Nanjing 210023, China; yxlnmr@nju.edu.cn (X.-L.Y.); yaoz@nju.edu.cn (Z.-J.Y.)
[6] State Key Laboratory of Pharmaceutical Biotechnology, Institute of Functional Biomolecules, School of Life Sciences, Nanjing University, Nanjing 210023, China; hmge@nju.edu.cn
[7] Division of Applied Thai Traditional Medicine, Faculty of Public Health, Naresuan University, Phitsanulok 65000, Thailand; orangejussamine@hotmail.com
* Correspondence: k_ingkaninan@yahoo.com; Tel.: +66-81-4817350

Supplementary Materials

Table S1: Calibration data, LOD, and LOQ of the flavonoids **1-6** analyzed by HPLC.

Table S2: Intra- and Inter-day precision and accuracy of flavonoids **1-6** analyzed by HPLC.

Table S1. Calibration data, LOD, and LOQ of the flavonoids **1-6** analyzed by HPLC.

Flavonoids	Linearity range (µg/ml)	Regression equation	Correlation coefficient (r^2)	LOD (µg/ml)	LOQ (µg/ml)
**1**	0.5 - 100	y = 23100x - 4376.5	0.9999	0.09	0.5
**2**	5.0 - 400	y = 20691x + 69562	0.9997	0.85	5.0
**3**	0.5 - 100	y = 64186x - 48477	0.9997	0.15	0.5
**4**	0.5 - 100	y = 27499x - 5199	0.9999	0.05	0.5
**5**	0.5 - 100	y = 65813x - 10389	0.9999	0.09	0.5
**6**	0.5 - 100	y = 44580x + 4643.1	0.9999	0.04	0.5

Table S2. Intra- and Inter-day precision and accuracy of flavonoids **1-6** analyzed by HPLC.

Flavonoids	Concentration levels (µg/ml)	Intra-day (*n*=3)		Inter-day (*n*=9)		Accuracy (*n*=3)	
		Measured concentration (µg/ml) ± SD	Precision (%RSD)	Measured concentration (µg/ml) ± SD	Precision (%RSD)	Recovery (%) Mean ± SD	Precision (%RSD)
**1**	3	2.71 ± 0.003	0.13	2.74 ± 0.05	1.83	90.04 ± 1.87	2.08
	25	23.63 ± 0.56	2.37	25.04 ± 0.73	3.00	86.69 ± 1.47	1.69
	65	62.94 ± 1.01	1.60	64.09 ± 1.04	1.63	90.05 ± 2.54	2.82
**2**	15	13.08 ± 0.24	1.86	13.01 ± 0.28	2.12	85.13 ± 1.53	1.79
	75	75.90 ± 1.23	1.62	77.15 ± 1.98	2.57	94.82 ± 0.73	0.77
	300	309.05 ± 1.41	0.46	311.04 ± 8.65	2.78	100.97 ± 2.44	2.42
**3**	3	2.94 ± 0.06	2.06	2.98 ± 0.09	2.85	106.17 ± 1.28	1.20
	25	27.01 ± 0.24	0.89	27.09 ± 0.61	2.25	98.46 ± 1.49	1.51
	65	66.27 ± 1.15	1.74	66.45 ± 1.64	2.47	109.92 ± 0.91	0.82
**4**	3	2.83 ± 0.07	2.61	2.87 ± 0.06	2.17	86.21 ± 2.40	2.78
	25	23.96 ± 0.56	2.33	24.48 ± 0.60	2.43	86.14 ± 2.06	2.40
	65	63.38 ± 1.26	1.99	64.74 ± 1.57	2.42	90.23 ± 0.57	0.63
**5**	3	2.58 ± 0.04	1.63	2.65 ± 0.06	2.18	103.43 ± 0.72	0.69
	25	25.47 ± 0.41	1.62	25.50 ± 0.63	2.46	100.67 ± 0.65	0.64
	65	64.63 ± 1.90	2.94	63.16 ± 1.64	2.49	105.74 ± 1.13	1.07
**6**	3	2.69 ± 0.02	0.90	2.70 ± 0.03	1.28	89.43 ± 1.27	1.42
	25	23.76 ± 0.49	2.06	24.26 ± 0.55	2.26	96.79 ± 2.19	2.27
	65	63.06 ± 1.12	1.77	64.24 ± 1.56	2.43	92.69 ± 0.83	0.89
